# Contrast-enhanced endoscopic ultrasonography differentiated pancreatic schwannoma from concurrent neuroendocrine tumor

**DOI:** 10.1007/s12328-026-02361-w

**Published:** 2026-06-09

**Authors:** Yumiko Yamashita, Keiji Hanada, Yasutaka Ishii, Akinori Shimizu, Tetsuro Hirano, Yasuhiro Okuda, Shuji Yonehara, Shinji Tanaka, Shiro Oka

**Affiliations:** 1 Department of Gastroenterology, Graduate School of Biomedical and Health Sciences, Hiroshima University Hospital, Hiroshima, Japan; 2 Department of Gastroenterology, Onomichi General Hospital, Hiroshima, Japan; 3 Department of Pathology, Onomichi General Hospital, Hiroshima, Japan; 4 Department of Gastroenterology, Graduate School of Biomedical and Health Sciences, Hiroshima University Hospital, Hiroshima University, 1-2-3 Kasumi, Minami-Ku, Hiroshima, 734-8551 Japan

**Keywords:** Pancreatic schwannoma, Pancreatic neuroendocrine tumor, Contrast-enhanced endoscopic ultrasonography

## Abstract

Pancreatic schwannomas are extremely rare and their variable imaging features on computed tomography (CT) and magnetic resonance imaging (MRI) make preoperative diagnosis challenging. A 73 year-old man was incidentally found to have a 13 mm hypoechoic mass in the pancreatic body on transabdominal ultrasonography. CT showed delayed enhancement, and MRI demonstrated hypointensity on T1, slight hyperintensity on T2, and hyperintensity on diffusion-weighted imaging (DWI). On endoscopic ultrasonography (EUS), the lesion appeared as a 13 mm hypoechoic mass, and contrast-enhanced EUS revealed gradual isoenhancement beginning 17 s after contrast injection. Another 6 mm hypoechoic lesion was identified in the pancreatic tail on EUS, and contrast-enhanced EUS showed rapid hyperenhancement beginning 12 s after contrast injection. EUS-guided fine-needle aspiration (EUS-FNA) was performed for both lesions, which were diagnosed as schwannoma and Grade 1 neuroendocrine tumor, respectively. A strategy of observation was adopted for both tumors. This is a rare case of concomitant pancreatic schwannoma and NET. EUS contributed to the detection of a small NET, while contrast-enhanced EUS revealed different vascular patterns between the two lesions, leading to a decision to perform EUS-FNA for both tumors.

**Supplementary Information:**

The online version contains supplementary material available at 10.1007/s12328-026-02361-w.

## Introduction

Schwannomas are tumors derived from Schwann cells of the peripheral nerve sheath, and they most commonly occur in the head, neck, and extremities. They are generally benign, and careful observation is acceptable when patients are asymptomatic and the lesion shows no tendency to enlarge [1, 2]. Primary pancreatic schwannomas are extremely rare, and it has been reported that they account for only about 0.03% of all pancreatic tumors [3]. Although pancreatic schwannomas have been reported in several cases, their variable appearances on computed tomography (CT) and magnetic resonance imaging (MRI) often complicate imaging assessment, making preoperative diagnosis challenging [4–7]. While endoscopic ultrasonography (EUS) has been described in several reports, the use of contrast-enhanced EUS remains rare [8, 9], and its imaging features have not been well established.

Here, we describe a rare case of concomitant pancreatic schwannoma and neuroendocrine tumor (NET). In this case, contrast-enhanced EUS enabled direct comparison of their enhancement patterns, leading to a definitive diagnosis by fine-needle aspiration (FNA).

## Case report

A 73-year-old man was found to have liver dysfunction during a medical checkup and underwent an abdominal ultrasound performed by his primary care physician. The transabdominal ultrasonography revealed a hypoechoic solid lesion approximately 13 mm in diameter in the pancreatic body, and he was referred to our department for further evaluation. His past medical history included hypertension and fatty liver. His father had prostate cancer; however, there was no family history suggestive of multiple endocrine neoplasia type 1 (MEN1). The patient did not experience any symptoms, and laboratory findings revealed a mildly elevated alanine aminotransferase level of 58 U/L. However, no elevation was observed in tumor markers such as carcinoembryonic antigen, carbohydrate antigen 19–9, neuron specific enolase, or pro-gastrin releasing peptide (Table 1).

**Table 1:** Laboratory findings

Hematology			Biochemistry					
WBC	6990	/μL	T-Bil	0.83	mg/dL	TP	7.6	g/dL
RBC	505	× 10^4^/μL	AST	28	U/L	Alb	4.7	g/dL
Hb	15.5	g/dL	ALT	58	U/L	T-Chol	195	mg/dL
Ht	47.8	%	LDH	203	U/L	TG	62	mmol/L
Plt	27.3	× 10^4^/μL	ALP	75	U/L	HDL-C	58.5	mmol/L
			r-GTP	41	U/L	Na	141	mmol/L
*Tumor markers*			AMY	48	U/L	K	4.2	mmol/L
CEA	1.3	ng/mL	P-AMY	24	U/L	Cl	104	mmol/L
CA19-9	22.3	U/mL	LIPA	40	U/L	Ca	9.5	mg/dL
NSE	11.1	ng/mL	BUN	17.0	mg/dL	CRP	0.02	mg/dL
Pro-GRP	34.4	pg/mL	Cr	0.86	mg/dL	intactPTH	37	pg/mL

**Fig. 1:**
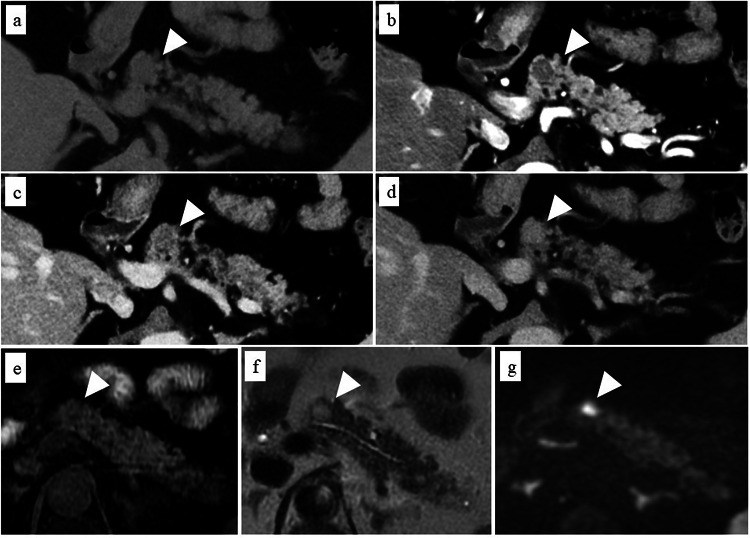
Computed tomography and Magnetic resonance imaging. **a**–**d**: The mass in the pancreatic body (arrow) on contrast-enhanced CT shows a gradually increasing enhancement pattern (**a**: unenhanced phase, **b**: arterial phase, **c**: portal phase, and **d**: equilibrium phase). **e**–**g**: MRI shows hypointensity on T1-weighted imaging (**e**), mild hyperintensity on T2-weighted imaging (**f**), and hyperintensity on diffusion-weighted imaging (**g**)

**Fig. 2:**
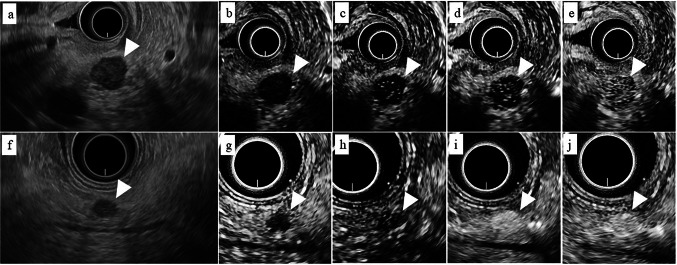
Endoscopic ultrasonography. **a**–**e**: The 13 mm mass in the pancreatic body (arrow) appears as a well-defined, round, hypoechoic lesion on B-mode imaging (**a**). On contrast-enhanced EUS, it shows gradual isoenhancement beginning at 17 s after contrast injection (**b**: 0 s, **c**: 17 s, **d**: 19 s, **e**: 60 s). **f**–**j**: The 6 mm mass in the pancreatic tail (arrow) appears as a well-defined, round, hypoechoic lesion on B-mode imaging (**f**). On contrast-enhanced EUS, it shows rapid hyperenhancement beginning at 12 s after contrast injection (**g**: 0 s, **h**: 12 s, **i**: 14 s, **j**: 60 s)

Contrast-enhanced computed tomography (CT) revealed a well-defined, round mass approximately 13 mm in diameter in the pancreatic body showing a delayed enhancement pattern (Fig. 1a–d). The lesion showed no enhancement in the arterial phase (Fig. 1b), but demonstrated enhancement similar to the pancreatic parenchyma from the portal venous (Fig. 1c) to the equilibrium phase (Fig. 1d). Magnetic resonance imaging (MRI) showed a hypointense signal on T1-weighted imaging (Fig. 1e), a slightly hyperintense signal on T2-weighted imaging (Fig. 1f), and a hyperintense signal on diffusion-weighted imaging (DWI) (Fig. 1g). Endoscopic ultrasonography (EUS) using a linear echoendoscope (GF-UE290, Olympus, Tokyo, Japan) and an ultrasound processor (ME-3, Olympus) demonstrated a well-defined, round hypoechoic mass measuring 13 mm in the pancreatic body (Fig. 2a). On contrast-enhanced EUS using perflubutane microbubbles (Sonazoid®, GE Healthcare), it showed gradual isoenhancement beginning at 17 s after contrast injection (Fig. 2b–e). Additionally, another lesion was identified in the pancreatic tail: a well-defined, round hypoechoic mass measuring 6 mm (Fig. 2f). This lesion was not detected on CT or MRI. On contrast-enhanced EUS, it showed rapid hyperenhancement beginning at 12 s after contrast injection with prolonged contrast retention (Fig. 2g–j). As the two masses demonstrated different enhancement characteristics on contrast-enhanced EUS, they were regarded as different tumors pathologically, and EUS-guided fine-needle aspiration (EUS-FNA) was performed for both lesions. For the pancreatic body mass, two passes were performed using a 22-gauge FNB needle (Trident®, Micro-Tech)—one with the suction technique and one with the slow-pull technique. For the pancreatic tail mass, one pass was performed using a 25-gauge FNB needle (TopGain®, Medico’s Hirata) with the slow-pull technique.

**Fig. 3:**
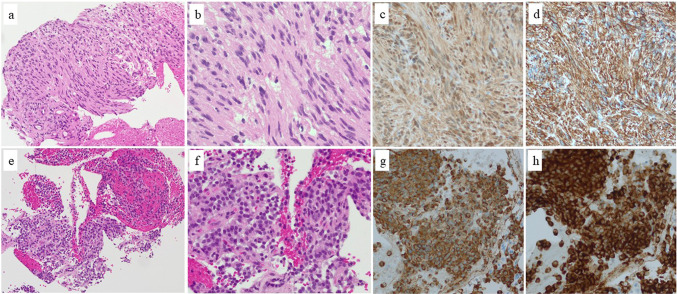
Pathological findings. a–d: The mass in the pancreatic body: **a** hematoxylin and eosin (H&E) staining, × 40; **b** H&E staining, × 400, consisting of spindle-shaped tumor cells arranged in a fascicular pattern; **c** positive for S-100; **d** positive for vimentin. e–h: The mass in the pancreatic tail: **e** H&E staining, × 40; **f** H&E staining, × 400, showing small tumor cells with finely granular, pale eosinophilic cytoplasm; **g** positive for chromogranin A; **h** positive for synaptophysin

On histopathological examination, the pancreatic body lesion consisted of spindle-shaped tumor cells arranged in a fascicular pattern on hematoxylin and eosin (HE) staining (Fig. 3a, b). On immunohistochemical analysis, it showed diffuse positivity for S-100 protein and vimentin (Fig. 3c, d), while CD34, c-kit, and desmin were negative. The Ki-67 labeling index was less than 1%. These findings are consistent with schwannoma corresponding to Antoni type A, which is characterized by higher cellularity. In contrast, the tail lesion showed small tumor cells with finely granular, pale eosinophilic cytoplasm on HE staining (Fig. 3e, f). On immunohistochemical analysis, it showed diffuse positivity for chromogranin A and synaptophysin (Fig. 3g, h). These findings are consistent with a neuroendocrine tumor (NET). No mitotic figures were observed, and the Ki-67 labeling index was < 3%, classifying the tumor as Grade 1 (G1), according to the 2022 WHO Classification of Endocrine and Neuroendocrine Tumours [10].

In summary, the pancreatic body lesion was diagnosed as a schwannoma, and the tail lesion as a NET G1. Given that most schwannomas are benign and the NET was G1 and less than 10 mm in size, a follow-up observation strategy was adopted. After one year, the lesions remain stable without any significant change, and surveillance is ongoing.

## Discussion

In prior reports of pancreatic schwannomas, the mean age at diagnosis was in the 50s, with a slight female predominance and they are frequently found in the pancreatic head and body [5–7]. Clinical symptoms include abdominal pain, diarrhea, nausea/vomiting, weight loss, and jaundice, although some cases are asymptomatic and incidentally detected [5–7]. Similarly, in our case, the patient was also asymptomatic, and the lesion was incidentally identified during a routine health checkup.

The CT findings of pancreatic schwannomas are variable, making differential diagnosis challenging. Previous studies report that accurate preoperative diagnosis is achieved in only 20–40% of cases [5, 6], with lesions often misinterpreted as pancreatic cystic neoplasms, solid pseudopapillary neoplasm, NET, pancreatic ductal adenocarcinoma, or acinar cell carcinoma. Characteristic CT features include well-defined lesions with low attenuation, cystic components, and delayed enhancement [4–7]. These imaging findings have been associated with histopathological subtype and tumor size. Antoni type A tumors, composed of densely packed spindle cells, typically appear as solid masses with mild enhancement, whereas Antoni type B lesions tend to be cystic and non-enhancing. Smaller tumors are more often solid and homogeneous, while larger lesions frequently display cystic degeneration and heterogeneous enhancement [4, 5, 7, 11, 12]. In our case, the lesion was small and Antoni type A, presenting as a well-defined, homogeneously enhancing solid mass—consistent with previously described patterns.

Several cases of EUS have been reported, with imaging findings diverse and heterogeneous, ranging from solid to cystic lesions, similar to findings on CT [8, 9, 13–18]. Reports of contrast-enhanced EUS in pancreatic schwannomas are extremely limited, with only two cases identified in the available literature. In one case, the lesion appeared as a cystic tumor with strong enhancement of the solid component [8]. In the other case, the lesion appeared as a solid tumor, and contrast-enhanced EUS showed short term contrast effects in the early phase and washed out quickly. Elastography was also performed in that case, demonstrating a strain ratio < 0.05, indicating a markedly stiff lesion [9]. In our case, the pancreatic tail NET began to enhance 12 seconds after contrast injection, and within 2 seconds it appeared as a hypervascular lesion, showing an enhancement pattern consistent with NET [19], whereas the pancreatic body schwannoma began to enhance 17 seconds after contrast injection, and even after 2 seconds, the contrast microbubbles were still gradually entering the lesion, giving the impression of a slow infiltration. Previous studies have reported delayed enhancement on contrast-enhanced CT [5, 6, 12, 14, 17, 18], and in our case, CT also demonstrated delayed enhancement from the portal venous to equilibrium phases, which was consistent with the enhancement pattern observed on contrast-enhanced EUS.

The difference in contrast uptake speed between the two tumors may reflect their vascular architecture and histological characteristics. Pancreatic NET generally have high microvessel density and rich intratumoral blood flow, resulting in rapid contrast distribution and early arterial-phase enhancement. In contrast, schwannomas tend to have relatively lower vascularity, and their dense (Antoni A) or edematous/degenerative (Antoni B) stroma may delay contrast penetration, leading to slower and weaker enhancement.

Although B-mode EUS demonstrated two similar-appearing lesions, CE-EUS revealed clearly different vascular enhancement characteristics. This finding raised suspicion that the lesions were not homogeneous multifocal tumors but represented distinct pathological entities. This contrast-dependent heterogeneity influenced our procedural strategy: rather than sampling a single representative lesion, we decided to perform EUS-FNA on both tumors. Thus, CE-EUS provided clinically meaningful information that supported targeted tissue acquisition and facilitated accurate pathological evaluation.

**Fig. 4:**
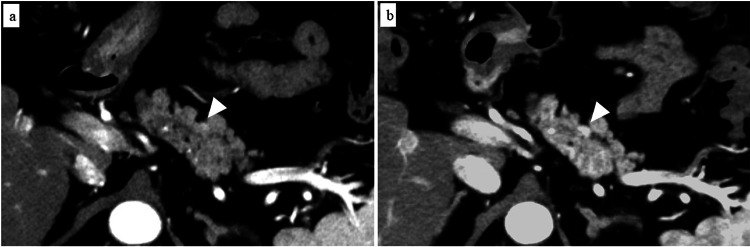
Contrast-enhanced CT images of the pancreatic tail lesion. **a**: Pancreatic parenchymal phase image obtained at the time of initial diagnosis. A very subtle hyperattenuating area is retrospectively visible at the site where the NET was later identified. **b**: Pancreatic parenchymal phase image obtained during follow-up. The lesion appears as a small contrast-enhanced nodular lesion in the pancreatic tail

In the present case, the 6-mm NET was not detected on the initial CT or MRI but was identified by EUS. CT was performed using a pancreatic dynamic protocol including the pancreatic parenchymal, portal venous, and equilibrium phases with a slice thickness of 2.5 mm, whereas MRI was performed with a slice thickness of 5 mm. The relatively larger slice thickness in MRI may have contributed to the lesion not being detected. On retrospective review of the initial CT images, a very subtle hyperattenuating area could be identified in the pancreatic parenchymal phase at the location where the NET was later confirmed in the pancreatic tail. However, this finding was extremely subtle and was considered too minimal to be confidently recognized as a lesion at the time of the initial evaluation (Fig. 4a). In contrast, on follow-up CT performed during observation, the lesion became more clearly visible as a contrast-enhanced nodular lesion (Fig. 4b). Although both examinations were obtained during the pancreatic parenchymal phase, slight variations in the timing of image acquisition relative to contrast enhancement may have influenced lesion conspicuity. The lesion might have been more clearly visualized if an earlier arterial phase had been obtained. Previous studies have suggested that EUS has a higher sensitivity than CT for detecting small pancreatic neuroendocrine tumors [22]. Therefore, EUS may play an important role in detecting small PNENs that are difficult to identify using cross-sectional imaging modalities. In the present case, EUS enabled the detection of a 6 mm lesion that was not clearly identified on CT or MRI.

In recent years, an increasing number of cases of pancreatic schwannoma have been reported in which a diagnosis could be established by EUS-FNA [12, 14–18, 20, 21]. However, there are also cases in which FNA failed to provide a diagnosis [23] or was not performed at all [4–9, 11, 13]. In cases where FNA was not performed, it is presumed that the presence of cystic components discouraged the procedure. In cases where no cellular material was obtained by FNA, it is conceivable that the tumor was punctured in an Antoni type B area, which is characterized by lower cellularity, thereby making cytological diagnosis difficult. In our case, the schwannoma was a contrast-enhanced solid tumor, and as the majority of the lesion was considered to be composed of Antoni A areas, sufficient tissue could be obtained by FNA to allow additional immunohistochemical staining.

Although approximately 90% of pancreatic schwannomas are benign, malignant cases have been reported, with suggestive features including large tumor size, irregular margins with internal heterogeneity, progressive enlargement, and invasion into surrounding organs [5–7]. In particular, regarding tumor size, one study reported that when a cutoff value of 6.9 cm was applied, the sensitivity and specificity were 71.4 and 75.4%, respectively [5]. In our case, the tumor was small, well-defined, and round in shape, without any evidence of invasive tendency. Considering its benign nature and the absence of symptoms, a strategy of follow-up observation was adopted. The NET in the pancreatic tail was less than 10 mm in size and classified as Grade 1, and was managed with the same observational approach. This strategy is consistent with Japanese clinical practice guidelines and previous reports, which permit surveillance for small, low-grade pancreatic NETs [24, 25]. Accurate diagnosis by contrast-enhanced EUS and EUS-FNA contributed to the treatment strategy, and the tumors remained stable during the short-term follow-up period of 1 year.

Regarding the coexistence of schwannoma and NET, no previous reports have described such an association, and the coexistence of the two tumors was considered likely incidental. Although genetic testing was not performed, the NET in this case appeared to be sporadic rather than MEN1-associated, because there was no family history of endocrine tumors, no biochemical or radiological evidence of parathyroid or pituitary disease, and the tumor was solitary. In addition, other genetic syndromes, including neurofibromatosis type 1 (NF1), were not clinically suspected because the pancreatic schwannoma was solitary and there were no characteristic clinical findings such as café-au-lait macules, multiple cutaneous neurofibromas.

Here, we report a case of pancreatic schwannoma concurrent with NET. EUS enabled the detection of a small NET that was not clearly identified on CT or MRI. In addition, contrast-enhanced EUS revealed different enhancement patterns between the two lesions, which played an important role in the differential diagnosis and led to the decision to perform EUS-FNA on both tumors. Accurate diagnosis guided management and allowed a strategy of observation to be adopted.

## Supplementary Information

Below is the link to the electronic supplementary material.

Supplementary Material 1.
