# Supplementary material for: Contrast-enhanced endoscopic ultrasonography differentiated pancreatic schwannoma from concurrent neuroendocrine tumor
Source: Clin J Gastroenterol. 2026 Jun 9;19(4):955–60. doi: 10.1007/s12328-026-02361-w (PMC13424386; doi:10.1007/s12328-026-02361-w)
Supplement: Supplementary file 1 — Supplementary Material 1. [file 12328_2026_2361_MOESM1_ESM.docx]

| Supplementary Table 1.  Imaging findings of pancreatic schwannomas according to histological subtype and tumor size | | |  |
| --- | --- | --- | --- |
|  |  |  |  |
| **Factor** | **Imaging Characteristics** | |  |
|  | **Morphology** | **Enhancement pattern** |  |
| Antoni type A | Solid | Homogeneous, mild |  |
| Antoni type B | Cystic or partially cystic | Poor or absent |  |
| Small tumors | Solid, well-defined | Homogeneous |  |
| Large tumors | Cystic degeneration, internal necrosis | Heterogeneous |  |

| Supplementary Table 2. Characteristics of Antoni Type A and Type B in Schwannomas | | |
| --- | --- | --- |
| **Feature** | **Antoni Type A** | **Antoni Type B** |
| Cellularity | High | Low |
| Cellular arrangement | Densely packed spindle cells  with palisading pattern | Loosely arranged cells  with indistinct palisading |
| Stromal features | Compact stroma | Myxoid or edematous stroma |
| Degenerative change | Minimal | Frequent cystic or degenerative changes |
